# Methodological Framework for a Multimodal Rat Model of Bleomycin-Induced Fibrosis and Autologous Tissue Grafting

**DOI:** 10.3390/mps9030094

**Published:** 2026-06-10

**Authors:** Razvan George Bogdan, Iulian-Alexandru Ciprian Blidisel, Ionut Ciobota, Anca Maria Campean, Alina Helgiu, Claudiu Helgiu, Ioan Catalin Bodea, Dan Ionel Orbulescu, Rodica Elena Heredea, Zorin Petrisor Crainiceanu

**Affiliations:** 1 Doctoral School, “Victor Babeș” University of Medicine and Pharmacy Timișoara, 300041 Timișoara, Romania; razvan.bogdan@umft.ro (R.G.B.); dan.orbulescu@umft.ro (D.I.O.); 2 County Clinical Emergency Hospital “Pius Brînzeu”, 300723 Timișoara, Romania; crainiceanu.zorin@umft.ro; 3 Department IX, General Surgery, Hepato-Bilio-Pancreatic Surgery Center, “Victor Babeș” University of Medicine and Pharmacy Timișoara, 300041 Timișoara, Romania; 4 “Pius Branzeu” Center for Training and Experimental Surgery, “Victor Babeș” University of Medicine and Pharmacy, 300041 Timișoara, Romania; ionut.ciobota@umft.ro; 5 Department of Microscopic Morphology/Histology, “Victor Babeș” University of Medicine and Pharmacy Timișoara, 300041 Timișoara, Romania; acimpeanu@umft.ro; 6 Center of Expertise for Rare Vascular Disease in Children, Emergency Hospital for Children “Louis Turcanu”, 300011 Timisoara, Romania; 7 Center of Genomic Medicine, “Victor Babeș” University of Medicine and Pharmacy Timișoara, 300041 Timișoara, Romania; 8 Research Center for Pharmaco-Toxicological Evaluation, “Victor Babeș” University of Medicine and Pharmacy Timișoara, 300041 Timișoara, Romania; 9 Faculty of Medicine, “Lucian Blaga” University of Sibiu, 550024 Sibiu, Romania; 10 County Clinical Emergency Hospital of Sibiu, 550245 Sibiu, Romania; 11 Department of Vascular Surgery, “Dr. Alexandru Augustin” Military Emergency Clinical Hospital, 550024 Sibiu, Romania; 12 Department of Surgery, “Iuliu Hatieganu” University of Medicine and Pharmacy, Croitorilor Street, No. 19–21, 400162 Cluj-Napoca, Romania; 13 Regional Institute of Gastroenterology and Hepatology “Octavian Fodor”, Croitorilor Street, No. 19–21, 400162 Cluj-Napoca, Romania; 14 Discipline of Clinical Practical Skills, Department I Nursing, Faculty of Medicine, “Victor Babeș” University of Medicine and Pharmacy Timișoara, 300041 Timișoara, Romania; 15 Plastic Surgery Department, “Victor Babeș” University of Medicine and Pharmacy Timișoara, 300041 Timișoara, Romania

**Keywords:** bleomycin-induced fibrosis, standardized experimental protocol, autologous tissue grafting, dermal–hypodermal remodeling preclinical imaging

## Abstract

Reproducible experimental models of localized dermal–hypodermal fibrosis are essential for standardized investigation of regenerative interventions. Variability in bleomycin dosing, anatomical targeting, and assessment strategies limits cross-study comparability. This study describes a methodological framework for standardized induction of early dermal–hypodermal remodeling in a rat model followed by autologous subcutaneous tissue grafting and multimodal longitudinal evaluation. Female Wistar rats underwent subcutaneous bleomycin administration at 1 mg/kg/day for three consecutive days. Clinical documentation, high-frequency ultrasonography with fixed imaging parameters, and sequential biopsies from a predefined thoracic anatomical site were performed at baseline, intermediate reassessment, and final evaluation. Autologous subcutaneous tissue grafting was conducted at Day 17 after study initiation. The protocol enabled controlled induction of early structural remodeling and consistent longitudinal documentation of dermal–hypodermal thickness, echogenicity changes, and histological architecture within a standardized anatomical region. This protocol development study establishes a reproducible and spatially consistent experimental platform integrating imaging and histological assessment, facilitating future hypothesis-driven investigations of fibrotic remodeling and regenerative strategies.

## 1. Introduction

Dermal and subcutaneous fibrosis is characterized by progressive extracellular matrix deposition, architectural distortion, and reduced tissue compliance [1,2,3,4]. In reconstructive surgery, particularly after radiotherapy, dermal–hypodermal remodeling alters tissue quality and vascularization, complicating graft integration and surgical planning [5,6,7]. Reliable preclinical models are essential for investigating fibrotic remodeling and evaluating regenerative strategies [8]. Bleomycin-induced cutaneous fibrosis is widely used in rodent models due to its reproducible capacity to trigger localized inflammatory activation followed by extracellular matrix deposition [9,10,11]. However, substantial variability in dosing regimens, anatomical targeting, sampling strategies, and imaging methodologies limits cross-study comparability and reproducibility. Standardized multimodal frameworks integrating imaging and histological assessment within the same anatomical region remain insufficiently defined. The present study describes a structured methodological framework for controlled induction of early dermal–hypodermal remodeling using a submaximal bleomycin protocol, followed by autologous subcutaneous tissue grafting and longitudinal multimodal evaluation. The aim is to establish a reproducible experimental platform emphasizing anatomical consistency, procedural standardization, and integrated structural assessment.

The present protocol is supported by previously generated imaging, clinical, and histological data obtained using the same experimental framework, confirming its capacity to produce reproducible and quantifiable remodeling patterns.

## 2. Experimental Design

This study was designed as a controlled, longitudinal experimental model aimed at standardizing the induction of dermal–hypodermal remodeling using bleomycin and its subsequent treatment through autologous subcutaneous tissue grafting in rats.

A total of 22 female Wistar rats were allocated into three experimental groups:

1. Treatment group (*n* = 15 initially), subjected to bleomycin-induced dermal–hypodermal remodeling followed by autologous subcutaneous tissue grafting;
2. Fibrosis control group (*n* = 5), subjected to bleomycin-induced remodeling without subsequent grafting;
3. Healthy reference group (*n* = 2), used for baseline full-thickness tissue sampling and euthanized immediately thereafter.

The experimental unit was the individual animal.

Animals in the healthy reference group were not included in longitudinal evaluation.

During postoperative monitoring, two animals from the treatment group reached predefined humane endpoints due to wound dehiscence and graft extrusion and were euthanized. The final number of animals included in longitudinal analysis of the treatment group was 13.

The experimental protocol followed a longitudinal within-subject design, with repeated clinical, ultrasonographic, and histological evaluations performed at predefined timepoints within the same anatomical region.

The sample size was determined based on feasibility considerations, ethical principles of reduction in animal research, and previously published bleomycin-induced fibrosis models. A formal sample size estimation was performed during study design as part of the ethical approval process. The longitudinal within-subject structure increases sensitivity by enabling repeated measurements in the same animal. The present study is not designed for statistical hypothesis testing or inter-group comparison. The healthy reference group plays a descriptive role in defining baseline dermal–hypodermal architecture and validating anatomical consistency of sampling. The primary analytical framework is based on longitudinal within-subject assessment, which reduces inter-individual variability and enables the detection of structural changes over time.

Sequential biopsies were performed within the same predefined anterior thoracic anatomical region to maintain spatial consistency across timepoints and ensure the reproducibility of tissue sampling.

Animals were allocated sequentially to predefined experimental groups according to planned group sizes at study initiation while maintaining comparable body weight distribution between groups. No additional selection criteria were applied.

The protocol comprises four sequential experimental stages. Stage 1 consists of baseline multimodal assessment, including standardized clinical photography, high-frequency ultrasonography with fixed imaging parameters, and Tru-Cut biopsy from a predefined anterior thoracic site. This stage requires approximately 5–7 min per animal.

Stage 2 consists of induction of early dermal–hypodermal remodeling through subcutaneous bleomycin administration at 1 mg/kg/day for three consecutive days. Each administration requires approximately 2–3 min per animal.

Stage 3 includes intermediate reassessment and surgical intervention performed 14 days after completion of bleomycin administration. Procedures include repeat imaging, biopsy, inguinal adipose tissue harvesting, mechanical processing, and subcutaneous graft placement. This stage requires approximately 12–15 min per animal under general anesthesia.

Stage 4 consists of final evaluation at Day 31, including standardized imaging and full-thickness excisional biopsy, followed by euthanasia. This stage requires approximately 7–9 min per animal.

The total duration of the protocol from baseline to final evaluation is 31 days.

The protocol requires the inclusion of a fibrosis control group receiving bleomycin without autologous grafting to differentiate spontaneous remodeling from graft-related effects. A healthy reference group may be included to establish baseline dermal–hypodermal architecture. Longitudinal within-subject assessment functions as an internal control by enabling repeated measurements within the same predefined anatomical site.

The model is optimized for the induction of early dermal–hypodermal remodeling and does not reproduce advanced chronic fibrosis. Histological evaluation is qualitative and does not include quantitative collagen morphometry or molecular characterization. Reproducibility depends on strict anatomical localization, operator consistency, and the maintenance of identical ultrasonographic acquisition parameters. Variations in injection depth or probe pressure may influence structural measurements.

Animals were monitored daily for general health status and signs of distress. Humane endpoints were predefined and included body weight loss exceeding 20% of baseline, persistent refusal of food or water for more than 48 h, severe signs of pain or distress, extensive necrosis or infection at the surgical site, or any irreversible clinical deterioration as assessed by the responsible veterinarian. In such cases, immediate humane euthanasia was performed according to the approved protocol.

Analgesic medication was not routinely administered in order to avoid potential interference with inflammatory and fibrotic pathways central to bleomycin-induced remodeling. Systemic analgesics, including non-steroidal anti-inflammatory drugs and opioids, may modulate inflammatory signaling, vascular responses, and fibroblast activity, potentially introducing biological variability. Animals were monitored daily for signs of pain or distress, including reduced mobility, abnormal posture, piloerection, decreased grooming behavior, reduced food intake, or vocalization upon handling. Local anesthetic administration was also considered; however, it was not routinely applied, as it would require an additional needle insertion at the same anatomical site and may induce local tissue distortion, edema, or microvascular changes that could influence ultrasonographic measurements and histological evaluation. The decision to avoid local anesthesia was based on the need to preserve local tissue integrity and microenvironmental conditions within the predefined sampling site, as repeated local injections may introduce cumulative mechanical and vascular alterations that could affect longitudinal comparability of ultrasonographic and histological findings. Any animal exhibiting sustained clinical signs of discomfort was evaluated by the attending veterinarian and predefined humane endpoints were applied when necessary. This approach was reviewed and approved as part of the institutional ethical authorization.

This study does not involve large-scale datasets deposited in publicly available repositories.

The present manuscript focuses on methodological standardization and procedural reproducibility rather than hypothesis-driven efficacy testing.

The protocol generates both quantitative variables, including dermal–hypodermal thickness measurements expressed in millimeters, and qualitative parameters derived from clinical, ultrasonographic, and histological evaluation. The longitudinal within-subject design enables structured comparison across predefined timepoints as well as between-group assessment while reducing inter-site variability. Detailed statistical analysis and outcome reporting will be presented in subsequent hypothesis-driven studies based on this methodological framework. The present article focuses on reproducibility, procedural integration, and technical consistency rather than treatment efficacy. In addition, the reproducibility of the protocol is supported by previously obtained datasets using the same experimental framework, demonstrating consistent induction of dermal–hypodermal remodeling and measurable structural changes across ultrasonographic, clinical, and histological domains. These data confirm that the protocol generates quantifiable and biologically relevant outcomes suitable for longitudinal analysis and comparative experimental applications.

All procedures were performed by a single experienced operator to minimize inter-operator variability.

### 2.1. Materials

1. Bleomycin sulfate, 15,000 IU/vial, powder for solution for injection (Accord Healthcare Polska Sp. z o.o., Warsaw, Poland; distributed by Accord Healthcare Ltd., North Harrow, UK, lot no: 62401252).
2. Sodium chloride 0.9% sterile solution for injection (B. Braun Melsungen AG, Melsungen, Germany).
3. Semi-automatic biopsy needle, VELOX 2, 14 G × 200 mm (Medax S.r.l., Poggio Rusco, MN, Italy; REF: VT14200-00; Lot no.: 04758-24).
4. Polypropylene monofilament suture, Optilene^®^, 4-0 (B. Braun Melsungen AG, Melsungen, Germany; sterile surgical suture).
5. Povidone–iodine 10% cutaneous solution, Betadine^®^ (Egis Pharmaceuticals PLC, Budapest, Hungary; antiseptic solution).
6. Neutral buffered formalin 10% solution, prepared by the hospital pharmacy (County Clinical Emergency Hospital “Pius Brînzeu”, Timișoara, Romania).
7. Ketamine hydrochloride injectable solution, veterinary formulation (Experimental Surgery and Training Center “Pius Brînzeu”, Timișoara, Romania).
8. Xylazine hydrochloride injectable solution, veterinary formulation (Experimental Surgery and Training Center “Pius Brînzeu”, Timișoara, Romania).
9. Sterile disposable surgical drapes (Experimental Surgery and Training Center “Pius Brînzeu”, Timișoara, Romania).
10. Disposable sterile syringe, 1 mL, 26G Luer Slip, SERIX (Changzhou Shuangma Medical Devices Co., Ltd., Changzhou, Jiangsu, China; imported by Medplaza Health SRL, Bucharest, Romania; sterile, single-use; LOT: 20240930).

### 2.2. Equipment

1. Vscan Air™ portable ultrasound system with 12 MHz linear probe (GE HealthCare, Chicago, IL, USA).
2. Aesculap Favorita II clipper (B. Braun, Tuttlingen, Germany).
3. Smartphone camera with integrated digital imaging system, iPhone 15 Pro (Apple Inc., Cupertino, CA, USA).
4. Battery-powered bipolar electrocautery (Experimental Surgery and Training Center “Pius Brînzeu”, Timișoara, Romania).
5. Automated heating pad system (Experimental Surgery and Training Center “Pius Brînzeu”, Timișoara, Romania).
6. Rectal temperature probe (Experimental Surgery and Training Center “Pius Brînzeu”, Timișoara, Romania).
7. Surgical instruments (Experimental Surgery and Training Center “Pius Brînzeu”, Timișoara, Romania).

### 2.3. Animals and Housing Conditions

Twenty-two adult female Wistar rats, 16 weeks of age and weighing 250–300 g at inclusion, were used in this study. Animals were housed in a specific pathogen-free facility within the Experimental Surgery and Training Center “Pius Brînzeu”, Timișoara, Romania, operating under veterinary sanitary authorization no. 815/11.06.2021.

Animals underwent a 7-day acclimatization period prior to the initiation of experimental procedures. During this period, animals were monitored daily for general health status and weighed upon arrival and immediately before study commencement.

Rats were housed in groups of 3–5 per cage under controlled environmental conditions. Ambient temperature was maintained at 22 °C with a 12 h light/12 h dark cycle. Standard laboratory chow and water were provided ad libitum. Environmental enrichment was provided throughout the study and included nesting material and structural cage elements.

All procedures complied with Directive 2010/63/EU and national legislation governing the protection of animals used for scientific purposes.

### 2.4. Histological Processing and Evaluation

All biopsy specimens were obtained from the same predefined anterior thoracic site. Immediately after harvesting, samples were fixed in 10% neutral buffered formalin.

Following fixation, specimens were processed using a standard paraffin-embedding protocol. Tissue blocks were sectioned at 4 µm thickness using a rotary microtome and mounted on glass slides.

Sections were stained with hematoxylin and eosin (H&E) according to standard laboratory procedures.

Histological evaluation was performed under light microscopy at ×10 and ×40 magnification by an investigator blinded to treatment group allocation.

The evaluation focused on qualitative assessment of dermal–hypodermal architecture, including

- Collagen bundle organization and density;
- Dermal thickening;
- Presence of inflammatory infiltrate;
- Vascular changes;
- Structural integrity of subcutaneous tissue.

The purpose of histological assessment was to document overall structural remodeling patterns of the dermal–hypodermal compartment rather than to perform quantitative collagen morphometry or molecular characterization.

## 3. Procedure

### 3.1. Baseline Multimodal Assessment (5–7 Min per Animal)

**Figure 1:**
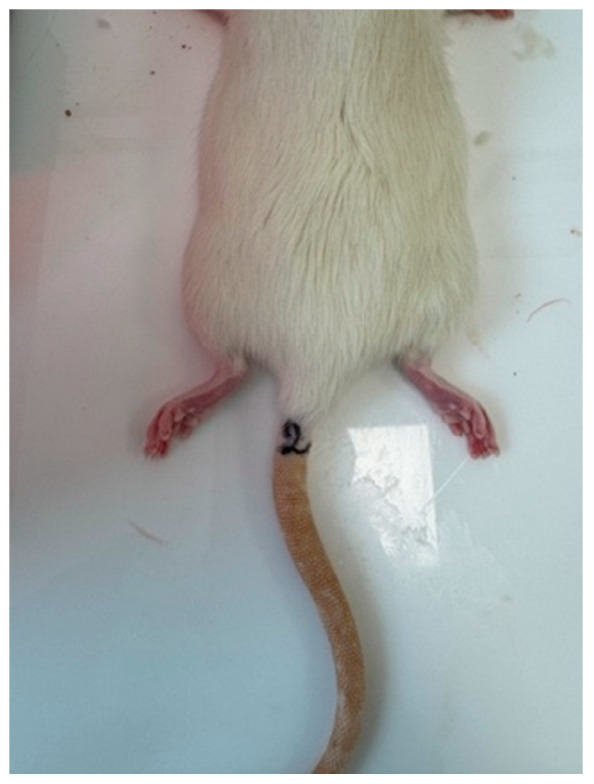
Individual animal identification by dorsal tail marking. Permanent skin marker labeling at the dorsal base of the tail was used for individual identification and longitudinal tracking of each animal throughout the experimental period.

**Figure 2:**
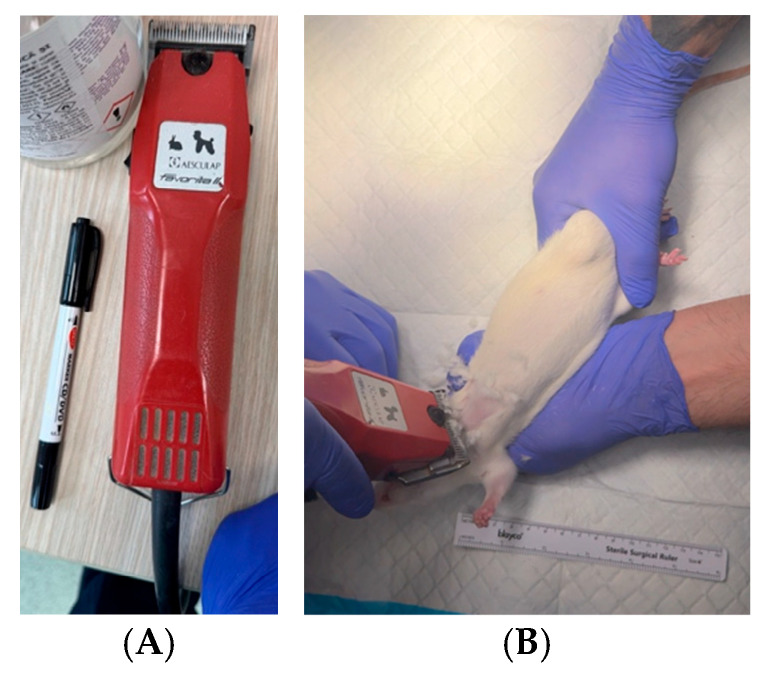
Preparation of the anterior thoracic region. (**A**) Shaving of the anterior left thoracic area using an Aesculap Favorita II clipper. (**B**) Thoracic mammary gland region after complete hair removal, prior to antiseptic preparation.

**Figure 3:**
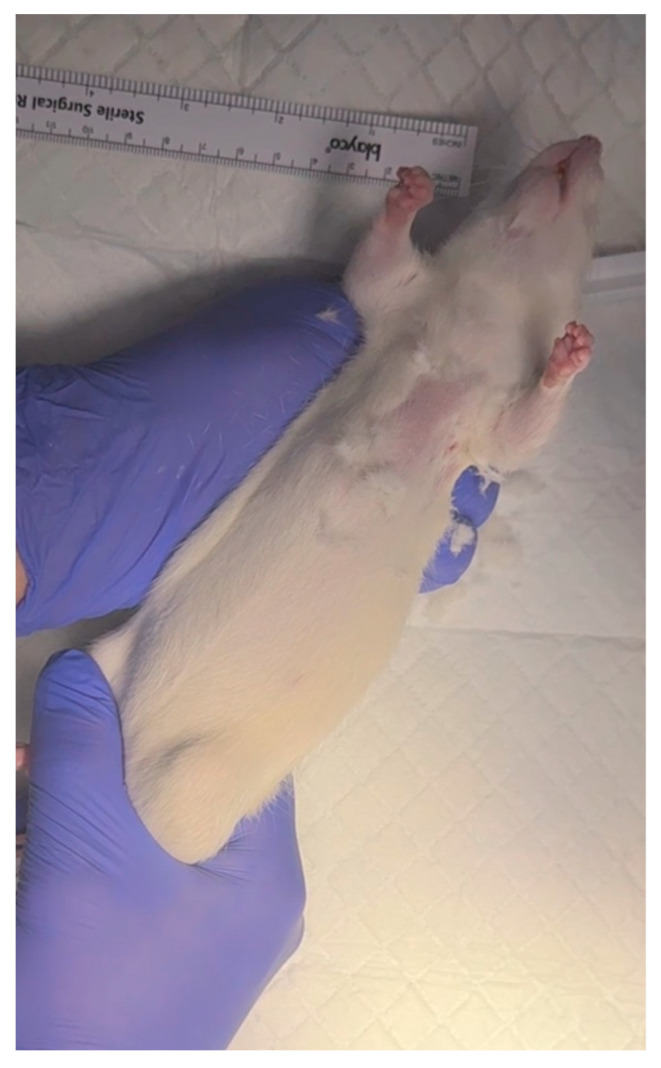
Baseline clinical documentation. Standardized anterior thoracic photographs obtained on Day 0 using a high-resolution digital camera for longitudinal clinical comparison.

**Figure 4:**
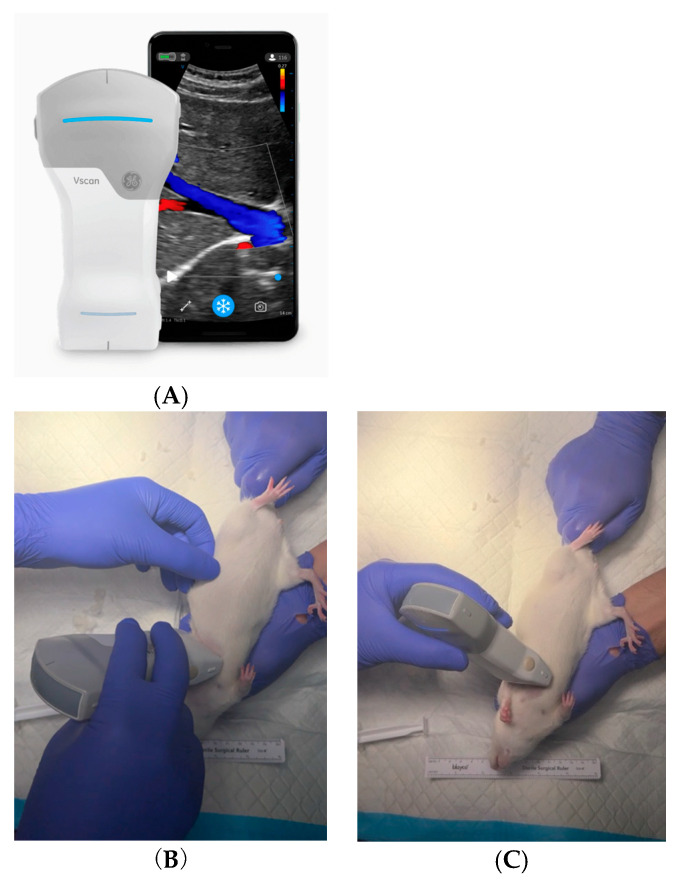
Ultrasonographic baseline assessment. (**A**) Vscan Air™ portable ultrasound system equipped with a 12 MHz linear probe. (**B**,**C**) Animal positioned in dorsal recumbency during imaging; gentle manual restraint was applied to ensure consistent probe placement and minimize motion artifacts during ultrasonographic evaluation.

**Figure 5:**
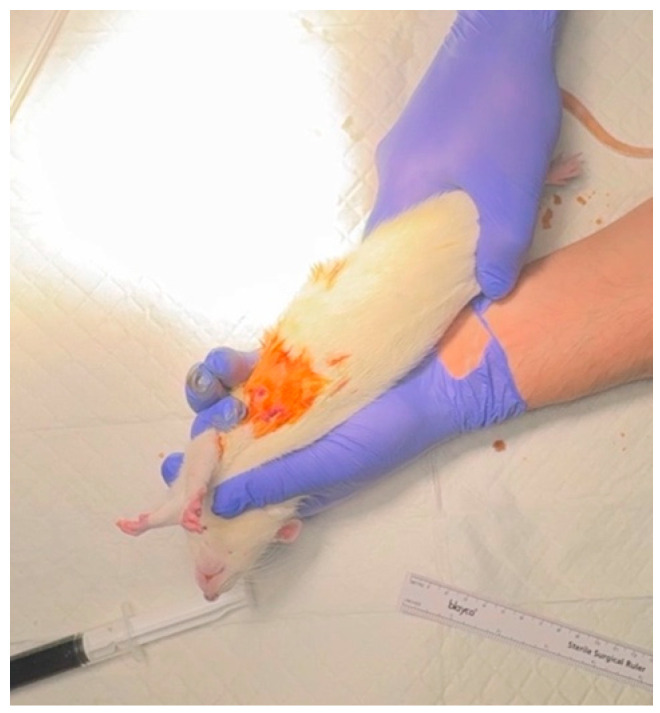
Antiseptic preparation of the anterior thoracic field. Disinfection of the shaved anterior thoracic region using povidone–iodine solution under sterile conditions prior to baseline assessment and intervention.

**Figure 6:**
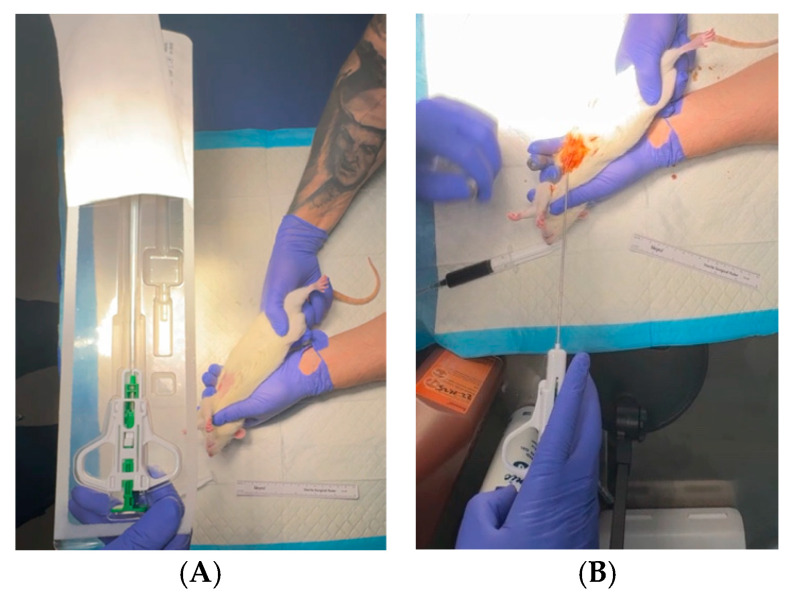
Baseline Tru-Cut biopsy procedure. (**A**) Semi-automatic 14 G biopsy needle (Tru-Cut^®^, Velox 2, 250 mm) used for tissue sampling. (**B**) Acquisition of baseline biopsy cores from the predefined anterior thoracic site.

**Figure 7:**
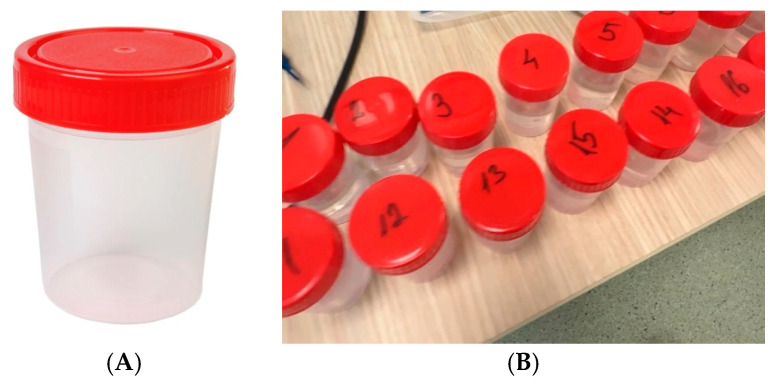
Sample fixation and container labeling. (**A**) Individual container filled with 10% buffered formalin for tissue fixation. (**B**) Numerical labeling on container caps used for sample identification.

1. Mark the dorsal base of the tail using a permanent skin marker to ensure individual identification (Figure 1).

2. Shave the anterior left thoracic region using an electric clipper (Figure 2A,B).

3. Position the animal in dorsal recumbency without anesthesia and apply gentle manual restraint to minimize motion artifacts during imaging.
4. Acquire standardized clinical photographs using a fixed distance and angle (Figure 3).

5. Apply acoustic gel and perform ultrasonographic assessment using a 12 MHz linear probe. Imaging settings, including gain, depth, and focal zone, were maintained constant throughout all examinations to ensure inter-timepoint comparability (Figure 4).

6. Disinfect the shaved area with 10% povidone–iodine solution (Figure 5).

7. Perform a Tru-Cut biopsy at the predefined thoracic site and collect 3–4 cores measuring approximately 4 mm in length and 1 mm in diameter (Figure 6).

8. Place biopsy specimens immediately in individually labeled containers containing 10% neutral buffered formalin (Figure 7).

9. Achieve hemostasis by local compression at the biopsy site.

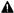


**CRITICAL STEP**

Maintain identical anatomical localization and probe positioning for all animals to ensure longitudinal reproducibility.

### 3.2. Induction of Dermal–Hypodermal Fibrosis (Days 1–3; 2–3 Minutes per Session)

**Figure 8:**
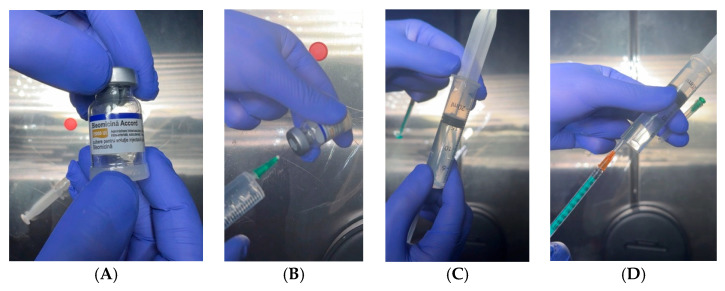
Preparation and handling of bleomycin solution. (**A**) Lyophilized bleomycin vial (15,000 IU). (**B**) Addition of 15 mL sterile 0.9% saline solution. (**C**) Reconstituted solution at a final concentration of 1 mg/mL. (**D**) Transfer of the solution into individual sterile 1 mL syringes (26 G Luer Slip) for subcutaneous administration.

1. Reconstitute bleomycin 15,000 IU with 15 mL sterile 0.9% saline to obtain a final concentration of 1 mg/mL (Figure 8A–C).

2. Prepare individual sterile 1 mL syringes for each animal (Figure 8D).
3. Calculate the injected volume according to body weight at 1 mg/kg. For animals weighing 250–300 g, the injected volume corresponded to approximately 0.25–0.30 mL per administration.
4. Inject the calculated volume subcutaneously into the predefined anterior thoracic site. The injection site was anatomically standardized as the region immediately inferior to the nipple within the shaved anterior thoracic field to ensure consistent localization across animals.
5. Repeat the administration once daily for three consecutive days.

The objective of this administration protocol was to induce an early, organized dermal–hypodermal remodeling response rather than advanced sclerotic fibrosis. This controlled submaximal induction was designed to simulate mild post-radiotherapy tissue alterations characterized by early extracellular matrix remodeling. The cumulative administered dose per animal was 3 mg/kg.

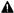


**CRITICAL STEP**

Confine the injection strictly to the subcutaneous plane to avoid intramuscular placement, which may produce heterogeneous remodeling.

### 3.3. Intermediate Reassessment and Autologous Grafting (Day 17; 12–15 Min per Animal)

**Figure 9:**
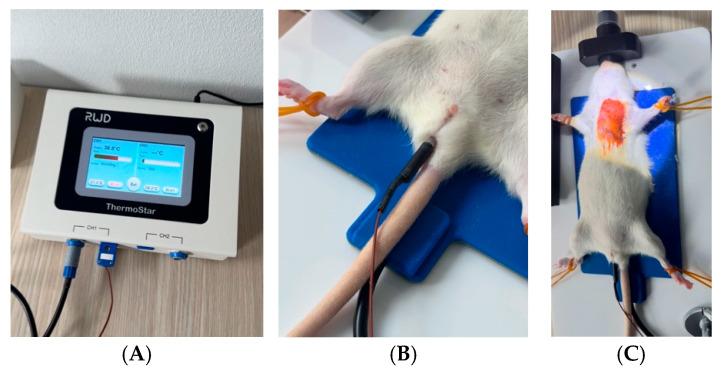
Intraoperative temperature monitoring and positioning. (**A**) Automated heating system used for maintenance of physiological body temperature. (**B**) Rectal temperature probe positioned for continuous monitoring. (**C**) Animal positioned on the heating pad during anesthesia.

**Figure 10:**
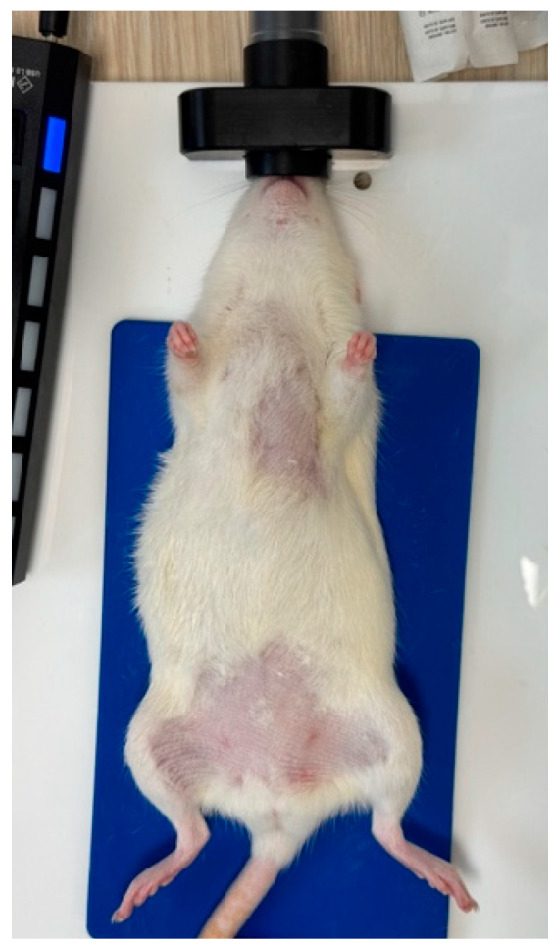
Preoperative clinical and ultrasonographic reassessment. Standardized clinical photographs and ultrasonographic evaluation performed prior to surgical intervention at the intermediate timepoint. Both the anterior thoracic and inguinal regions were re-shaved and prepared for the subsequent procedure.

**Figure 11:**
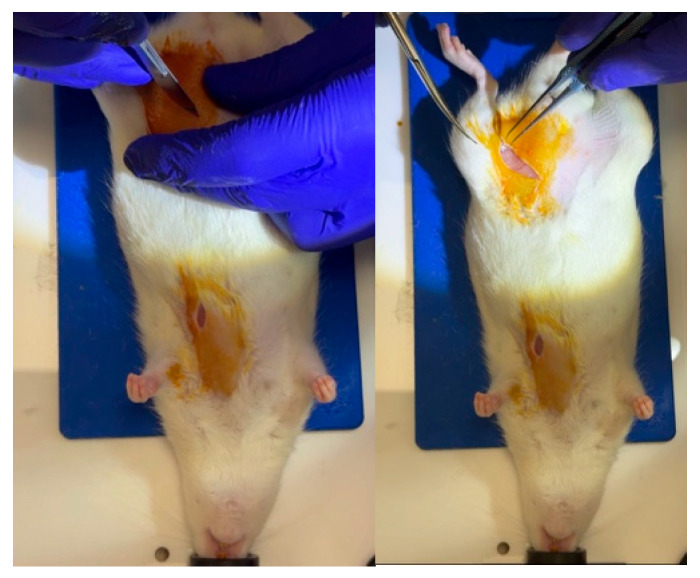
Inguinal incision for adipose tissue harvesting. Skin incision performed at the inguinal fold in the treatment group to access subcutaneous adipose tissue for graft harvesting.

**Figure 12:**
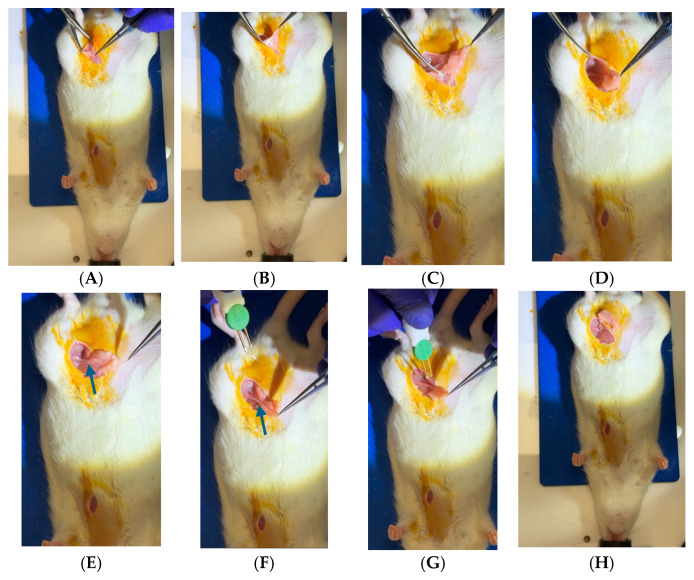
Inguinal adipose tissue harvesting procedure. (**A**–**D**) Blunt dissection using Stevens scissors to expose and mobilize autologous subcutaneous adipose tissue. (**E**–**G**) Identification (blue arrows), isolation, and bipolar coagulation of small vascular branches supplying the adipose tissue to achieve hemostasis. (**H**) Harvested inguinal adipose tissue prior to processing.

**Figure 13:**
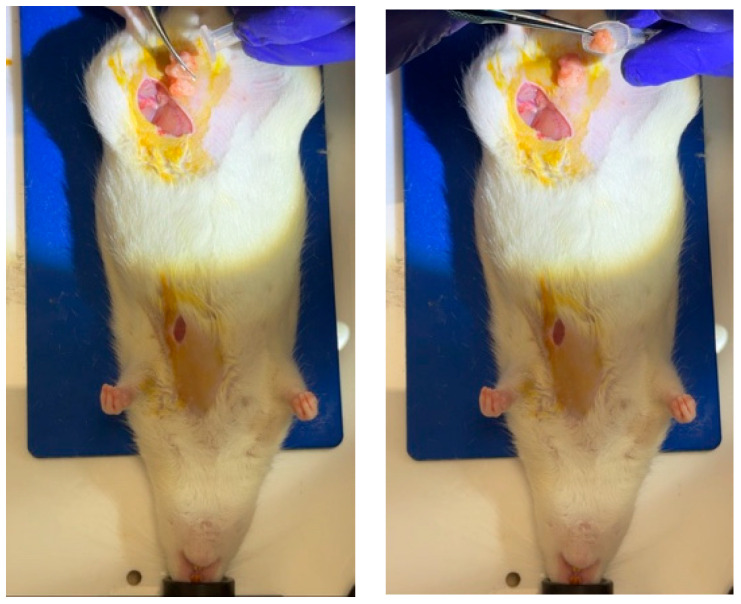
Processing of harvested adipose tissue. Harvested adipose tissue rinsed with sterile saline to remove blood residues and mechanically fragmented into small pieces without enzymatic processing, followed by transfer into a sterile 1 mL syringe for graft placement.

**Figure 14:**
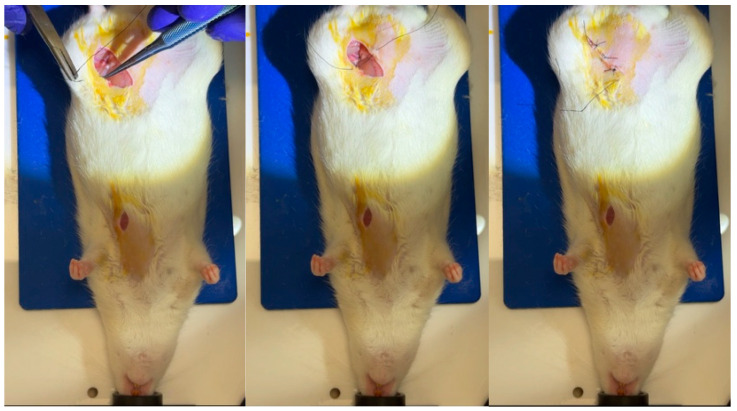
Closure of the inguinal donor site. Inguinal donor site closed using 4-0 polypropylene sutures following adipose tissue harvesting.

**Figure 15:**
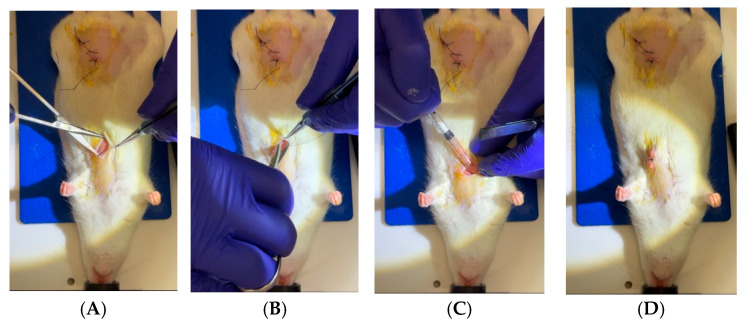
Placement of the adipose graft in the thoracic recipient site. (**A**,**B**) Minimal blunt dissection of the thoracic subcutaneous tissue to create a recipient pocket. (**C**) Introduction of the processed adipose tissue into the subcutaneous plane through the pre-existing Tru-Cut incision. (**D**) Closure of the thoracic recipient site using 4-0 polypropylene sutures.

1. Induce general anesthesia using ketamine 100 mg/kg and xylazine 10 mg/kg intraperitoneally.
2. Confirm adequate depth of anesthesia by absence of withdrawal reflex.
3. Maintain body temperature using a heating pad (Figure 9).

4. Provide oxygen supplementation via face mask throughout the procedure.
5. Re-shave and disinfect thoracic and inguinal regions.
6. Perform clinical and ultrasonographic reassessment using identical parameters (Figure 10).

7. Obtain a Tru-Cut biopsy from the predefined thoracic site.
8. Perform an inguinal skin incision (Figure 11).

9. Dissect bluntly to expose subcutaneous adipose tissue (Figure 12A–H).

10. Identify and coagulate small vascular branches using bipolar electrocautery.
11. Harvest adipose tissue. The harvested adipose tissue was collected as free, non-vascularized graft material and was not transferred as a pedicled or vascularized flap.
12. Rinse the harvested tissue with sterile saline to remove residual blood and debris.
13. Mechanically fragment the tissue using sterile surgical scissors into small, uniform fragments without enzymatic processing.
14. Transfer fragmented tissue into a sterile 1 mL syringe using sterile microsurgical forceps, followed by gentle advancement of the plunger to facilitate controlled graft delivery (Figure 13).

15. Create a minimal recipient pocket through the existing Tru-Cut incision.
16. Close donor site using 4-0 polypropylene sutures (Figure 14).
17. Inject 0.4–0.6 mL of adipose tissue in multiple micro-deposits within a single subcutaneous plane (Figure 15C).
18. Close recipient sites using 4-0 polypropylene sutures (Figure 15D).

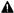


**CRITICAL STEP**

Ensure meticulous hemostasis before graft placement to prevent hematoma formation and graft extrusion.

### 3.4. Postoperative Monitoring (Daily; 1–5 Min per Animal)

1. Inspect wound integrity.
2. Assess mobility, grooming behavior, and food intake.
3. Apply predefined humane endpoints when necessary.

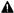


**CRITICAL STEP**

Monitor closely for self-inflicted wound dehiscence, which may lead to graft loss.

### 3.5. Final Assessment (Day 31; 7–9 Min per Animal)

**Figure 16:**
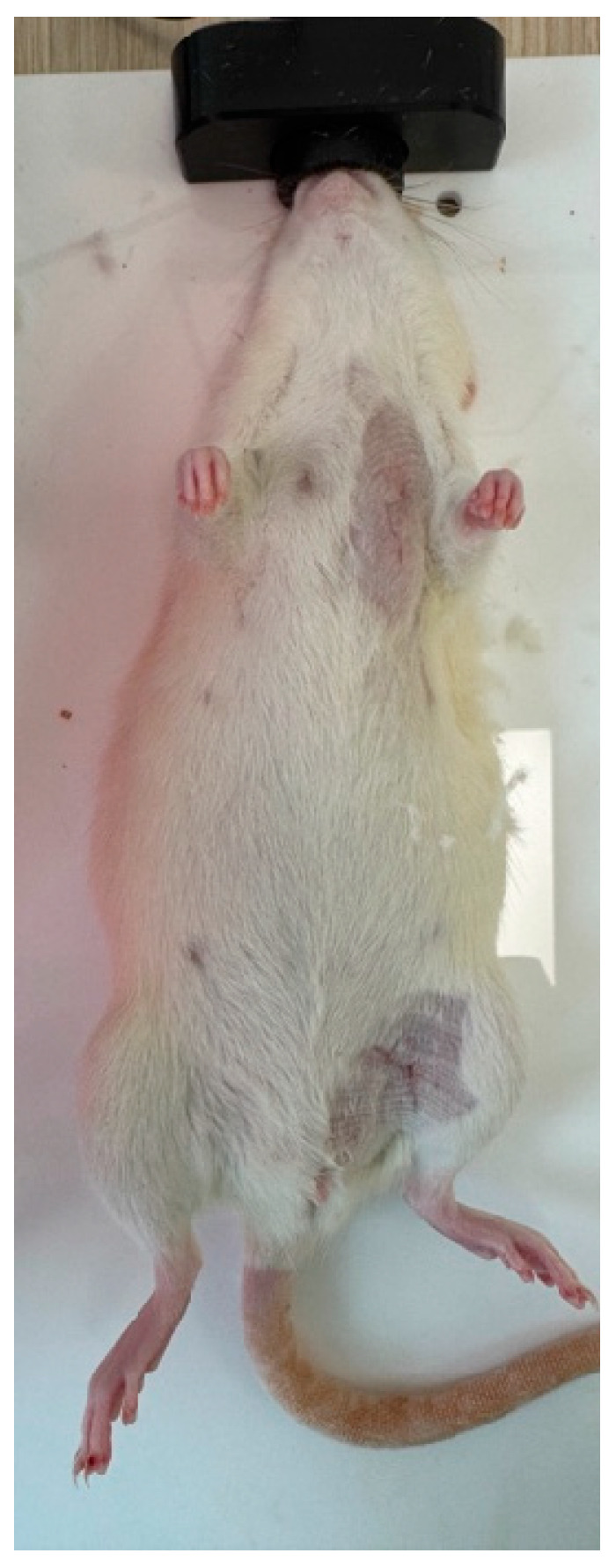
Final clinical appearance at Day 31. Anterior thoracic region at the final assessment (Day 31), demonstrating the integration of the autologous adipose graft within the previously bleomycin-treated field.

1. Re-shave the thoracic region.
2. Acquire standardized clinical photographs (Figure 16).

3. Perform final ultrasonographic assessment using the same imaging system and acquisition parameters described previously.
4. Excise a full-thickness specimen including skin and subcutaneous tissue down to fascia.
5. Fix specimens immediately in 10% neutral buffered formalin.
6. Perform euthanasia by cervical dislocation under deep anesthesia, carried out by trained personnel in accordance with institutional and legal guidelines.

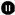


**PAUSE STEP**

After fixation, tissue samples may be stored in formalin at room temperature for up to 48 h before paraffin processing.

The chronological sequence of all experimental procedures, predefined assessment timepoints, and corresponding interventions is summarized in Table 1.

## 4. Expected Results

Successful induction of early dermal–hypodermal remodeling is expected to produce measurable structural changes at both ultrasonographic and histological levels.

**Figure 17:**
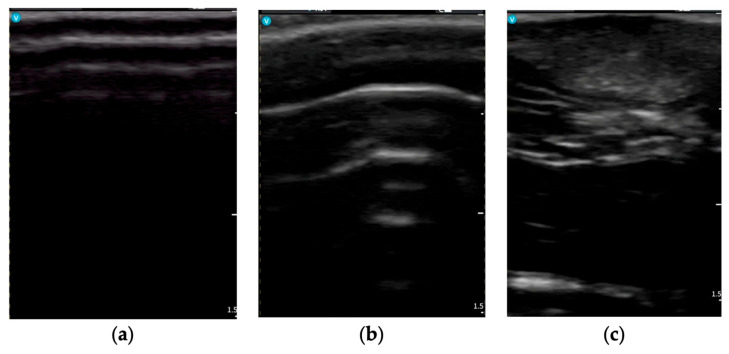
Representative longitudinal ultrasonographic images across predefined timepoints [12]. (**a**) Baseline (Day 0): preserved dermal–hypodermal architecture with homogeneous hypoechoic subcutaneous layer and fine echogenic septa. (**b**) Day 17: increased echogenicity, septal thickening, and reduced structural homogeneity consistent with early fibrotic remodeling. (**c**) Day 31: partial attenuation of echogenicity and septal prominence with focal hypoechoic areas corresponding to graft integration.

At Day 17, ultrasonography should demonstrate increased dermal–hypodermal thickness compared to baseline values, accompanied by mild to moderate changes in tissue echogenicity (Figure 17). The subcutaneous layer may appear more heterogeneous relative to baseline imaging. Tissue echogenicity was classified as hypoechoic, isoechoic, or hyperechoic relative to adjacent reference tissue. All examinations were digitally recorded to allow subsequent blinded review by an experienced soft-tissue ultrasonographer.

**Figure 18:**
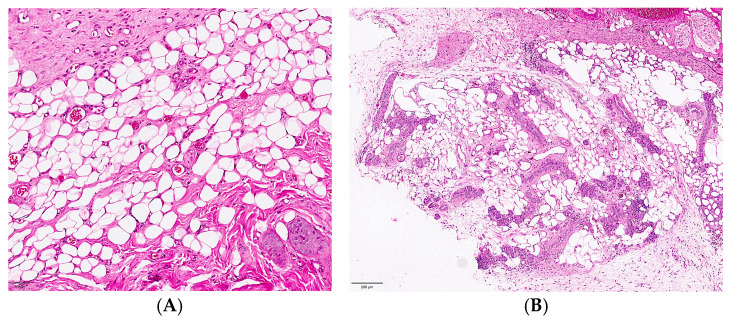
Representative histological features of dermal–hypodermal remodeling. (**A**) Day 17: early fibrotic remodeling characterized by increased collagen deposition, thickened connective septa, adipocyte distortion, and vascular dilation. (**B**) Day 31: heterogeneous tissue architecture following autologous grafting, with coexistence of viable adipose tissue, fibrotic septa, and areas of adipocyte degeneration.

Histological evaluation at the intermediate timepoint is expected to reveal early architectural remodeling characterized by increased collagen bundle density, dermal thickening, mild inflammatory infiltrate, and subtle vascular changes without extensive sclerosis (Figure 18A).

Following autologous subcutaneous tissue grafting, ultrasonographic assessment at Day 31 should demonstrate discrete hypoechoic or heterogeneous areas corresponding to graft integration within the subcutaneous plane (Figure 18B). Successful graft retention is associated with preserved subcutaneous architecture without large fluid collections or extensive necrosis. Representative ultrasonographic images illustrating these structural changes across timepoints are provided in Figure 17. Representative histological patterns corresponding to these stages are illustrated in Figure 18.

Deviations from the standardized procedure may result in the following imaging or structural findings:

- Absence of measurable structural remodeling following bleomycin administration, including lack of dermal–hypodermal thickening or echogenic alteration, suggesting insufficient subcutaneous localization;
- Diffuse intramuscular echogenic alteration indicating incorrect injection depth;
- Large anechoic collections consistent with hematoma formation;
- Loss of graft material secondary to wound dehiscence;
- Extensive necrosis at the recipient site.

Dermal–hypodermal thickness should be analyzed as the mean of three perpendicular measurements at each timepoint. Longitudinal within-subject comparison allows for the evaluation of structural remodeling dynamics. Echogenicity and tissue homogeneity should be interpreted descriptively and correlated with histological findings. Histological analysis should focus on architectural remodeling patterns rather than quantitative morphometry in the present protocol framework. Correlation between ultrasonographic findings and histological architecture is recommended to validate imaging-based assessment of dermal–hypodermal remodeling. Measurements should be performed at the same predefined anatomical location using identical probe positioning and acquisition settings to ensure longitudinal comparability.

Clinically, composite scores reflecting erythema, edema, tissue elasticity, and mobility increased from 0 at baseline to 9.28 ± 1.75 after bleomycin exposure and decreased to 7.50 ± 1.35 following grafting, demonstrating partial functional improvement consistent with imaging findings.

In a representative implementation of this protocol using the same experimental framework, ultrasonographic evaluation demonstrated a reproducible increase in dermal–hypodermal structural alteration following bleomycin administration, with mean semi-quantitative scores increasing from 0 at baseline to approximately 2.15 ± 0.58 at Day 17, confirming consistent induction of early remodeling. Following autologous subcutaneous tissue grafting, scores decreased to approximately 1.50 ± 0.50 at Day 31, indicating partial structural normalization. Quantitative ultrasonographic analysis showed an increase in dermal thickness from approximately 0.94 mm at baseline to 1.39 mm at Day 17, followed by a decrease after grafting, supporting measurable structural dynamics across timepoints. Structural changes should be interpreted based on within-subject comparison across timepoints, using baseline values as reference for detecting increases in thickness, changes in echogenicity, and alterations in tissue organization [12].

Histological evaluation confirmed these observations, showing increased collagen deposition, septal thickening, fibroblast proliferation, and vascular changes at the intermediate timepoint, followed by a heterogeneous remodeling pattern after grafting, characterized by coexistence of viable adipose tissue, fibrotic septa, and areas of active tissue reorganization.

## 5. Reagent Setup

Bleomycin Reconstitution

Reconstitute bleomycin sulfate 15,000 IU vial with 15 mL sterile 0.9% sodium chloride solution to obtain a final concentration of 1 mg/mL. Gently invert the vial until complete dissolution. Avoid vigorous shaking to prevent foam formation.

Prepare individual sterile 1 mL syringes immediately after reconstitution. Use the solution on the same day of preparation.

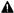


**CRITICAL STEP**

Ensure complete dissolution before withdrawal to avoid dosing variability.

Storage

Store unreconstituted vials according to the manufacturer’s instructions at controlled room temperature. Do not store reconstituted solution beyond the same working day.

10% Neutral Buffered Formalin

Use commercially prepared or hospital pharmacy-prepared 10% neutral buffered formalin for tissue fixation.

Immerse biopsy specimens immediately after harvesting. Maintain a minimum fixative-to-tissue volume ratio of 10:1.

Storage

Store formalin at room temperature in a closed container according to institutional safety regulations.

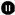


**PAUSE STEP**

Fixed tissue samples may remain in formalin for up to 24–48 h prior to paraffin embedding.

0.9% Sodium Chloride Solution

Use sterile 0.9% sodium chloride solution at room temperature for

- Bleomycin reconstitution;
- Rinsing harvested adipose tissue;
- Mechanical graft preparation.

Use single-use sterile containers and discard remaining solution after each session.

## 6. Patents

The authors declare that no patents have resulted from the work reported in this manuscript.

## 7. Discussion and Limitations

### 7.1. Rationale for Bleomycin Dose and Experimental Timeline

Bleomycin was selected due to its reproducible capacity to induce localized dermal and subcutaneous fibrotic remodeling in rodent models [9,10]. While classical protocols frequently rely on prolonged or repeated administration to generate dense sclerotic fibrosis, the present framework intentionally employed a submaximal regimen.

Administration of 1 mg/kg/day for three consecutive days was designed to initiate controlled profibrotic signaling and early extracellular matrix deposition without progressing toward advanced, end-stage fibrosis. The objective was to obtain an organized early dermal–hypodermal remodeling pattern rather than mature scar architecture.

The 14-day interval between the final bleomycin injection and surgical intervention was selected to allow transition from acute inflammatory response toward early structural remodeling. This timing provides a stabilized but non-terminal fibrotic substrate suitable for evaluating graft integration within an active remodeling environment rather than in chronically established scar tissue.

### 7.2. Strengths of the Multimodal Longitudinal Assessment

A major strength of the proposed framework is the integration of standardized clinical documentation, high-frequency ultrasonography, and sequential histological sampling across structured timepoints. This design enables longitudinal structural assessment while maintaining spatial consistency.

High-frequency ultrasonography provides repeatable measurement of dermal–hypodermal thickness and qualitative evaluation of tissue echogenicity using fixed imaging parameters and constant anatomical landmarks [9]. Maintaining identical probe positioning and acquisition settings across timepoints reduces operator-dependent variability and improves intra-study comparability.

Sequential biopsies obtained from the same predefined thoracic site ensure temporal continuity of histological assessment. By combining non-invasive imaging with controlled tissue sampling, the model allows progressive monitoring of structural remodeling without relying exclusively on terminal endpoints.

This integrated approach enhances methodological reproducibility and facilitates standardized evaluation of graft placement and tissue response within a controlled fibrotic substrate. Autologous fat grafting has been increasingly associated with modulation of scar architecture, improvement of tissue elasticity, angiogenesis, and reduction in fibrotic remodeling [13].

### 7.3. Methodological Limitations

Several methodological considerations should be acknowledged. The bleomycin protocol was intentionally designed to induce early dermal–hypodermal remodeling rather than advanced, dense fibrosis. Consequently, the model does not replicate the full architectural complexity of chronic radiation-induced scarring. Chronic fibrotic tissues also involve complex biomechanical alterations and mechanotransduction pathways that are not fully reproduced within early remodeling models [14].

Sequential biopsies were obtained from the same predefined anatomical region to ensure spatial consistency. Although this strategy enhances longitudinal comparability, localized sampling may introduce minor procedural tissue alterations.

Histological evaluation was limited to hematoxylin and eosin staining, focusing on structural assessment rather than quantitative collagen morphometry or molecular characterization. While sufficient for documenting architectural remodeling patterns, additional staining techniques could provide further matrix-specific detail in future applications of the model [15]. Future applications of this framework may also benefit from advanced dermatologic ultrasonography techniques for the assessment of superficial tissue architecture and structural remodeling [16].

The limited size of the healthy reference group represents a constraint in capturing the full spectrum of physiological variability. However, within the present protocol, this group plays a descriptive role for baseline structural characterization rather than statistical comparison, as the primary analytical framework relies on longitudinal within-subject assessment.

Finally, this study was conducted in a single species within a controlled preclinical setting. The framework is intended for methodological standardization and experimental reproducibility, and extrapolation to complex human fibrotic conditions should be approached cautiously.

## Figures and Tables

**Table 1:** Chronological organization of experimental procedures. Overview of predefined timepoints and corresponding interventions performed during the study, including clinical documentation, ultrasonographic evaluation, sequential biopsies, bleomycin administration, autologous adipose tissue grafting, and final histological sampling.

Day	Experimental Phase	Procedures Performed
0	Baseline Assessment	Shaving of anterior thoracic and inguinal regions; tail marking; standardized clinical photography; high-frequency ultrasonographic evaluation; Tru-Cut biopsy (3–4 cores); fixation of samples in 10% buffered formalin
1–3	Induction of Fibrosis	Subcutaneous bleomycin administration (1 mg/kg/day) at predefined anterior thoracic site; individualized dosing according to body weight
Day 17	Intermediate Assessment and Surgical Intervention	Re-identification and re-shaving; standardized photography and ultrasonography; Tru-Cut biopsy; general anesthesia; inguinal adipose tissue harvest; saline rinsing and mechanical fragmentation; transfer to sterile 1 mL syringe; minimal blunt undermining of thoracic subcutaneous tissue; placement of graft through existing Tru-Cut incision; closure of inguinal and thoracic incisions with 4-0 polypropylene sutures; postoperative monitoring
Postoperative Day 1	Early Postoperative Monitoring	Daily assessment of wound integrity and signs of distress; humane euthanasia of two animals due to wound dehiscence and graft extrusion
31	Final Assessment	Re-identification and re-shaving; standardized photography and ultrasonography; full-thickness excisional biopsy; fixation in 10% buffered formalin; euthanasia under deep anesthesia

## Abbreviations

The following abbreviations are used in this manuscript:

H&E	Hematoxylin and Eosin
IU	International Units
MHz	Megahertz
EU	European Union
